# Metformin Increases Cortisol Regeneration by 11βHSD1 in Obese Men With and Without Type 2 Diabetes Mellitus

**DOI:** 10.1210/jc.2016-2069

**Published:** 2016-07-26

**Authors:** Anna J. Anderson, Ruth Andrew, Natalie Z. Homer, Gregory C. Jones, Kenneth Smith, Dawn E. Livingstone, Brian R. Walker, Roland H. Stimson

**Affiliations:** University/British Heart Foundation Centre for Cardiovascular Science (A.J.A., R.A., N.Z.H., G.C.J., K.S., D.E.L., B.R.W., R.H.S), University of Edinburgh, Edinburgh EH16 4TJ, Scotland, United Kingdom; Diabetes Centre, Gartnavel General Hospital (G.C.J.), Glasgow, Scotland, United Kingdom; and Division of Medical Sciences and Graduate Entry Medicine (K.S.), School of Medicine, University of Nottingham, Royal Derby Hospital Centre, Derby, United Kingdom

## Abstract

**Context:**

The mechanism of action of metformin remains unclear. Given the regulation of the cortisol-regenerating enzyme 11βhydroxysteroid dehydrogenase 1 (11βHSD1) by insulin and the limited efficacy of selective 11βHSD1 inhibitors to lower blood glucose when co-prescribed with metformin, we hypothesized that metformin reduces 11βHSD1 activity.

**Objective:**

To determine whether metformin regulates 11βHSD1 activity in vivo in obese men with and without type 2 diabetes mellitus.

**Design:**

Double-blind, randomized, placebo-controlled, crossover study.

**Setting:**

A hospital clinical research facility.

**Participants:**

Eight obese nondiabetic (OND) men and eight obese men with type 2 diabetes (ODM).

**Intervention:**

Participants received 28 days of metformin (1 g twice daily), placebo, or (in the ODM group) gliclazide (80 mg twice daily) in random order. A deuterated cortisol infusion at the end of each phase measured cortisol regeneration by 11βHSD1. Oral cortisone was given to measure hepatic 11βHSD1 activity in the ODM group. The effect of metformin on 11βHSD1 was also assessed in human hepatocytes and Simpson-Golabi-Behmel syndrome adipocytes.

**Main Outcome Measures:**

The effect of metformin on whole-body and hepatic 11βHSD1 activity.

**Results:**

Whole-body 11βHSD1 activity was approximately 25% higher in the ODM group than the OND group. Metformin increased whole-body cortisol regeneration by 11βHSD1 in both groups compared with placebo and gliclazide and tended to increase hepatic 11βHSD1 activity. In vitro, metformin did not increase 11βHSD1 activity in hepatocytes or adipocytes.

**Conclusions:**

Metformin increases whole-body cortisol generation by 11βHSD1 probably through an indirect mechanism, potentially offsetting other metabolic benefits of metformin. Co-prescription with metformin should provide a greater target for selective 11βHSD1 inhibitors.

## Abstract (precis)

Obese men with and without type 2 diabetes received 28 days of metformin or placebo then underwent a deuterated cortisol infusion to measure 11βHSD1 activity. Metformin increased whole body 11βHSD1 activity in both groups.

Metformin is the mainstay of treatment in obese patients with type 2 diabetes mellitus (T2DM), yet the mechanism of action remains unclear. Metformin lowers glucose concentrations in part by suppressing hepatic gluconeogenesis (1), an effect thought to be primarily mediated through inhibition of the respiratory-chain complex I with subsequent activation of AMPK (2). Additional mechanisms contributing to the glucose-lowering effect of metformin have been proposed, such as the organic cation transporter Oct1, which enhances the action of metformin in the liver, whereas metformin may antagonize the effects of glucagon (reviewed in Ref. 3). A further potential molecular target for metformin action has been identified after the discovery of altered tissue cortisol regulation in obesity and T2DM (4–6).

Although circulating cortisol is controlled centrally by the hypothalamic-pituitary-adrenal (HPA) axis, tissue glucocorticoid levels are further regulated by the 11β-hydroxysteroid dehydrogenase (11βHSD) enzymes. The type 2 isozyme (11βHSD2) converts cortisol to inactive cortisone, modulating activation of mineralocorticoid receptors in relevant tissues such as kidney (7). The type 1 isozyme (11βHSD1) is more abundant across metabolically active tissues, particularly in the liver and adipose tissue, and primarily converts cortisone to cortisol (8). Transgenic mice overexpressing 11βHSD1 in adipose tissue or liver develop features of the metabolic syndrome such as obesity, glucose intolerance, and dyslipidemia (9, 10). In human obesity, hepatic 11βHSD1 activity is decreased whereas adipose tissue 11βHSD1 is increased, resulting in similar whole body cortisol regeneration by 11βHSD1 compared to lean individuals (4, 5, 11). In contrast, in obesity-associated T2DM, cortisol regeneration by 11βHSD1 in the whole body is increased while hepatic 11βHSD1 activity is unchanged compared with lean nondiabetic individuals (6, 12); because insulin suppresses hepatic 11β-HSD1 activity (13), the impaired insulin signaling associated with T2DM may drive the lack of suppression of hepatic 11βHSD1 in this group. These results highlight the potential benefit of inhibiting 11βHSD1 as a novel treatment for obesity-associated T2DM.

Numerous selective 11βHSD1 inhibitors have been developed (reviewed in Ref. 14); however, results from the published phase 2 trials have been disappointing. The vast majority of patients participating in these trials were co-prescribed metformin. We hypothesized that the improvement in insulin sensitivity induced by metformin may decrease hepatic 11βHSD1 activity and limit the efficacy of 11βHSD1 inhibition. Therefore, we tested whether metformin regulates cortisol regeneration by 11βHSD1 in obese individuals with T2DM (the target group for selective 11β-HSD1 inhibitors) and in obese euglycemic individuals (who have suppressed hepatic 11β-HSD1 unlike those with T2DM), using a deuterated cortisol infusion to measure whole-body 11βHSD1 activity (15).

## Subjects and Methods

### In vivo study protocol

Eight obese nondiabetic (OND) men and eight obese men with T2DM (ODM) were recruited to this double-blind, placebo-controlled crossover study. Inclusion criteria were: body mass index (BMI) ≥30 kg/m^2^; age, 18–70 years; alcohol intake <21 U per week; no exogenous glucocorticoid exposure in the preceding 6 months; normal screening blood tests (full blood count, kidney, liver, and thyroid function, and normal glucose in the OND group); <5% change in body weight over the preceding 3 months; not on any medications known to regulate cortisol metabolism (eg, antifungals, 5α-reductase inhibitors, or opiates); glycated hemoglobin (HbA1c) <10% (86 mmol/mol) if diet controlled or <8% (64 mmol/mol) if on metformin monotherapy (ODM group only). Informed consent was obtained from all participants, and approval was obtained for this study from the local research ethics committee. ODM participants remained on their other prescribed medications (eg, statins, antihypertensives) throughout the study. Participants were randomized to receive 28 days of either placebo or metformin 1 g twice daily; to account for any confounding effect of improving glycemic control on 11βHSD1, the ODM group underwent a third phase taking the sulfonylurea gliclazide 80 mg twice daily. There was a 3-day washout period between phases.

**Figure 1.**
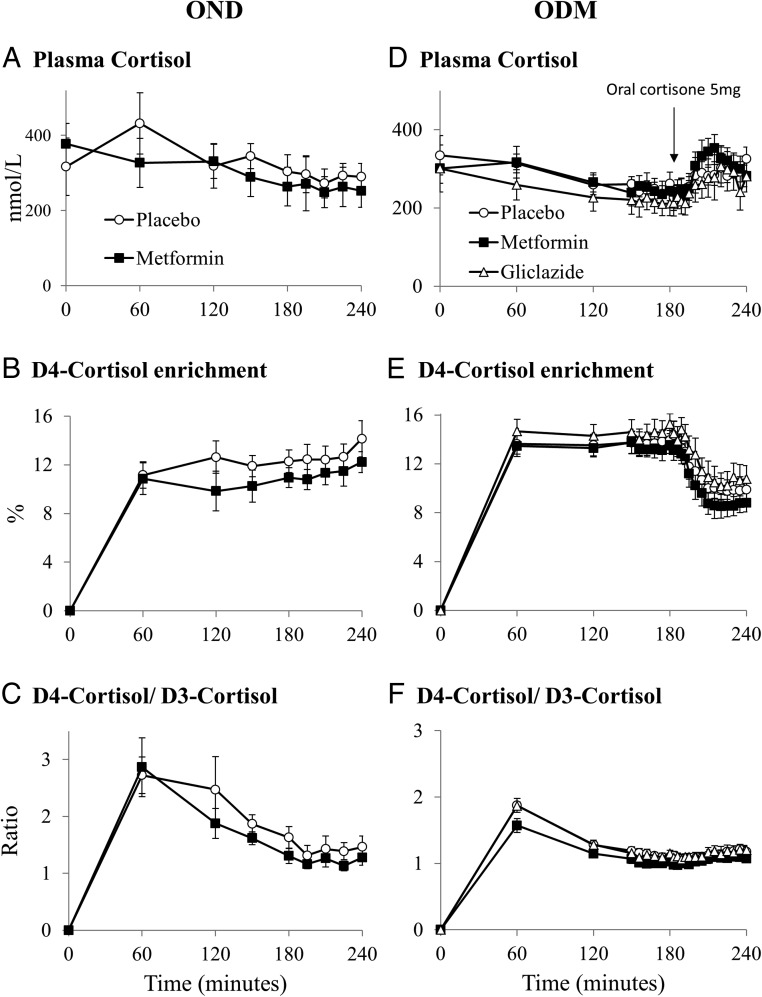
Endogenous and tracer cortisol measurements. Data are expressed as mean ± SEM for metformin (black squares), gliclazide (open triangles), and placebo (open circles). A and D, Plasma cortisol; B and E, D4-cortisol enrichment; C and F, D4-cortisol/D3-cortisol ratio in OND (n = 8; A–C) and ODM (n = 8; D–F) groups.

At the end of each phase, subjects attended the Clinical Research Facility at 8:30 am after an overnight fast. Measurements of height and weight were performed, and baseline blood samples were taken for fasting glucose, insulin, and HbA1c. To measure whole-body 11βHSD1 activity, cortisol (containing 20% 9,11,12,12-[^2^H]_4_-cortisol [D4-cortisol]; Cambridge Isotopes) was infused at 1.74 mg/h for 4 hours after an initial 3.5-mg bolus (16). In brief, D4-cortisol is converted to 9,12,12-[^2^H]_3_-cortisone (D3-cortisone) by 11βHSD2 due to the loss of the deuterium on the 11th carbon. D3-cortisone is then regenerated to D3-cortisol by 11βHSD1. Once in steady state, dilution of D4-cortisol by D3-cortisol is a specific measure of cortisol regeneration by 11βHSD1. Blood samples were taken at regular intervals once steady state was achieved (t + 150 minutes) (Figure 1). In the ODM group, after samples had been collected for steady-state measurements, oral cortisone (5 mg) was given at 180 minutes, and conversion to cortisol was measured over the next hour to determine hepatic 11βHSD1 activity (6).

### Effects of metformin on 11βHSD1 activity in vitro

Human primary hepatocytes (Bioreclamation IVT) were cultured according to the manufacturer's instructions. Human Simpson-Golabi-Behmel syndrome preadipocytes were cultured as previously described (17). Three days after plating (hepatocytes) or after completion of differentiation on day 12 (adipocytes), cells were cultured for 24 hours in vehicle or 100 nm, 1 μm, 10 μm, 100 μm, 1 mm, or 10 mm metformin hydrochloride (Sigma). Thereafter, cells were incubated with medium containing 1 μm cortisone (enriched with 20 nm 1,2-[^3^H]_2_-cortisone; GE Healthcare) for either 120 (hepatocytes) or 240 minutes (adipocytes) at 37°C to measure conversion to cortisol.

### Laboratory analysis

#### Biochemical measurements

Plasma glucose was measured using a colorimetric assay and insulin was measured by immunoassay using Abbott Architect analysers. HbA1c was measured by HPLC (HA8180 analyzer, Menarini Diagnostics). Endogenous and tracer glucocorticoids (cortisol, D4-cortisol, D3-cortisol, cortisone, and D3-cortisone) were measured by liquid chromatography tandem mass spectrometry as previously described (6).

Metformin and gliclazide were extracted from plasma (100 μL) using an SLE+ plate (Biotage) after enrichment with D6-metformin and D3-glyburide as internal standards (200 ng). Calibration standards ranged from 0.5–1000 ng. Analytes were eluted, reduced to dryness under nitrogen (40°C), and reconstituted in water/acetonitrile (100 μL; 80:20, v/v). Analysis was carried out by liquid chromatography tandem mass spectrometry. Chromatographic separation was on an ACE Excel Super2C18 column (100 × 3 mm; 2 μm) protected by a Kinetex KrudKatcher (Phenomenex) and detected on a 5500 QTrap (Seiex) operated by selective reaction monitoring in positive electrospray ionization mode. The mobile phase was 0.1% formic acid in water (A) and 0.1% formic acid in acetonitrile (B) at 0.2 mL/min and 30°C. Gradient elution was from 20–90% in B, where metformin and D6-metformin eluted at 1.1 minutes and gliclazide and D3-glyburide eluted at 2.0 and 2.3 minutes, with a total run time of 5 minutes. Transitions monitored for were *m/z* 130.1 → 60.1 and *m/z* 136.2 → 60.1 for metformin and D6-metformin, respectively, and *m/z* 324.2 → 153.1 and *m/z* 497.1 → 372.1, for gliclazide and D3-glyburide, respectively.

#### Analysis of tritiated steroids

Tritiated steroids were extracted from 200 μL of medium using methanol. [^3^H]_2_-Cortisone and [^3^H]_2_-cortisol were quantified by HPLC with online β-scintillation counting (Berthold LB509 detector; Berthold Technologies). Samples were analyzed in quadruplicate. Total protein was measured in each sample using the DC protein assay (Bio-Rad), and cortisol production rates were normalized for protein content.

### Cortisol kinetics

Cortisol kinetics were calculated as previously described (6). Steady-state samples were collected from 150 to 240 minutes in the OND group and from 150 to 180 minutes (time of cortisone ingestion) in the ODM group. Rate of appearance (Ra) of endogenous cortisol in the whole body during steady state was calculated using Equation 1:

(1) $$\text{Ra}\;\text{Cortisol}=\frac{\text{D}4-\text{Cortisol}\;\text{infusion}\;\text{rate}}{\text{D}4-\text{Cortisol}\;\text{Cortisol}\;\text{ratio}\;\text{rate}}-\text{Cortisol}\;\text{infusion}\;\text{rate}$$

Ra D3-cortisol (a specific measure of cortisol regeneration by 11βHSD1) was calculated using Equation 2:

(2) $$\text{Ra}\;\;\text{D}3-\text{Cortisol}=\frac{\text{D}4-\text{Cortisol}\;\text{infusion}\;\text{rate}}{\text{D}4-\text{Cortisol}/\text{D}3-\text{Cortisol}\;\text{ratio}}$$

Clearance of D4-cortisol was calculated by dividing the D4-cortisol infusion rate by the steady state D4-cortisol concentration. The Ra of cortisol after oral cortisone ingestion (a measure of hepatic 11βHSD1 activity) in the ODM group was calculated using Steele's non-steady state equation (Equation 3) where t denotes time, V is the volume of distribution, C(t) is the total cortisol concentration at time (t), and E(t) is the tracer to tracee ratio (D4 cortisol/cortisol). The volume of distribution for cortisol was taken as being 12 L, as in previous studies (12, 18).

(3) $$\text{Ra}\;\text{Cortisol}=\left(\frac{\text{D}4-\text{Cortisol}\;\text{infusion}\;\text{rate}}{\text{E}\left(\text{t}\right)}\right)-\left(\frac{\text{V}\times \frac{\text{C}\left(\text{t}\right)}{1+\text{E}\left(\text{t}\right)}\times \frac{\text{dE}\left(\text{t}\right)}{\text{dt}}}{\text{E}\left(\text{t}\right)}\right)$$

### Statistical analysis

Data are presented as mean ± SEM. Power calculations were performed using prior data indicating that the difference in the response of matched pairs was normally distributed with standard deviation 1.21 (16). Eight subjects per group provided >85% power to detect a 10% difference in the Ra of D3-cortisol with a 0.05 probability of a type I error associated with this test. Data were analyzed using SPSS version 21 (SPSS, Inc). Data were normally distributed using Kolmogorov-Smirnov testing. Comparisons between two related samples were performed using paired *t* tests and between three or more related samples using repeated measures ANOVA with post hoc Fisher's least significance difference (LSD) testing. Comparisons between two unrelated samples were performed using unpaired *t* tests. *P* < .05 was considered significant.

## Results

### Anthropometric and biochemical data

Subjects in the ODM group were older and had higher fasting glucose and HbA1c than the OND participants (Table 1). BMI was not different between the two groups (*P* > .2), and body weight did not change between phases (data not shown). One of the OND subjects developed transient diarrhea during the metformin phase. No other side effects were reported by any of the participants. Metformin and gliclazide decreased fasting glucose to a similar extent in the ODM group, with similar trends in HbA1c, but metformin did not alter fasting glucose in OND participants (Table 1). Metformin and gliclazide were only detected in the plasma during the appropriate phases (data not shown).

**Table 1.** Anthropometric and Biochemical Data

	OND		ODM		
	Placebo	Metformin	Placebo	Metformin	Gliclazide
Age, y	43.6 ± 4.6	43.6 ± 4.6	65.8 ± 0.8^b^	65.8 ± 0.8	65.8 ± 0.8
BMI, kg/m^2^	37.4 ± 2.6	37.3 ± 2.8	34.2 ± 1.1	34.2 ± 1.2	34.6 ± 1.1
Fasting glucose, mmol/L	5.6 ± 0.6	5.3 ± 0.2	10.8 ± 1.0^b^	7.7 ± 0.5^a^	7.0 ± 0.6^a^
HbA1c, %/mmol/mol	5.7 ± 0.2 (38.8 ± 2.0)	5.7 ± 0.2 (38.4 ± 2.2)	7.2 ± 0.2 (55.4 ± 2.5)^c^	6.9 ± 0.3 (52.4 ± 3.0)	7.1 ± 0.3 (53.6 ± 3.6)
Fasting insulin, mU/L	17.1 ± 5.1	10.6 ± 2.2	24.2 ± 9.7	24.9 ± 10.3	21.4 ± 7.5
HOMA-IR	5.0 ± 1.9	2.5 ± 0.6	9.4 ± 3.2	7.0 ± 2.2	7.2 ± 1.7
Total cholesterol, mmol/L	4.5 ± 0.4	4.7 ± 0.6	3.5 ± 0.2^b^	3.4 ± 0.2	3.4 ± 0.2
D4-Cortisol clearance, L/min	0.5 ± 0.1	0.6 ± 0.1	0.5 ± 0.1	0.5 ± 0.1	0.6 ± 0.1

Abbreviation: HOMA-IR, homeostasis model of assessment for insulin resistance. Data are expressed as mean ± SEM for data from OND (n = 8) and ODM (n = 8) participants. Phases were compared using paired *t* tests in the OND group and repeated measures ANOVA with post hoc Fisher's LSD testing in the ODM group. Placebo-phase data for OND and ODM groups were compared using unpaired *t* tests.

a *P* < .05 vs placebo;

b *P* < .05;

c *P* < .01 vs OND group.

### Cortisol kinetics

Fasting cortisol was similar between OND and ODM groups and was unaltered by metformin or gliclazide treatment (Figure 1, A and D).

#### Steady-state measurements

**Figure 2.**
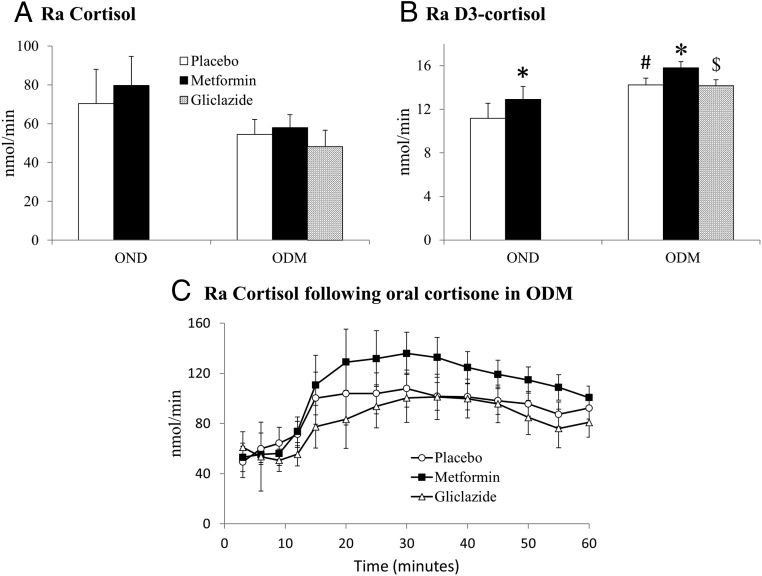
The effect of metformin on 11βHSD1 activity in vivo. Data are expressed as mean ± SEM for the effect of metformin (black columns), gliclazide (bricked columns), and placebo (white columns) on the Ra of cortisol (A) and D3-cortisol (B) during steady state. C, The effect of metformin (black squares), gliclazide (open triangles), and placebo (open circles) on Ra cortisol after 5 mg oral cortisone ingestion in the ODM group. Phases were compared using paired *t* tests in the OND group and repeated measures ANOVA with post hoc Fisher's LSD testing in the ODM group. Placebo-phase data in OND and ODM groups were compared using the unpaired *t* test. *, *P* < .05 vs placebo; $, *P* < .05 vs metformin; #, *P* < .05 vs OND group.

Steady-state D4-cortisol enrichment was achieved after 150 minutes of D4-cortisol infusion in both groups (Figure 1, B and E). Metformin increased the Ra of D3-cortisol (Ra D3-cortisol, a specific measure of whole-body 11βHSD1 activity) compared with placebo (both groups) and gliclazide (ODM group only) (Figure 2B). Ra D3-cortisol was higher in ODM compared with OND participants. The Ra of cortisol (Figure 2A) and rate of D4-cortisol clearance (Table 1) were unaltered by either treatment and were not different between ODM and OND groups.

#### Non-steady-state measurement of hepatic 11βHSD1 activity

Cortisol production by hepatic 11βHSD1 was calculated using Equation 3 in the ODM group. Metformin tended to increase Ra cortisol after oral cortisone (*P* = .07) (Figure 2C).

### Effect of metformin on 11βHSD1 activity in vitro

**Figure 3.**
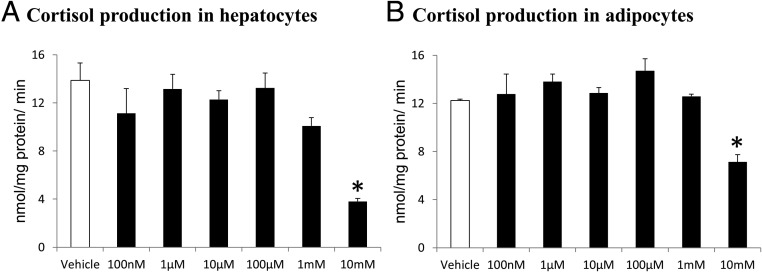
The effect of metformin on 11βHSD1 activity in vitro. Data are expressed as mean ± SEM for the rate of cortisol production in primary human hepatocytes (A) and human Simpson-Golabi-Behmel syndrome adipocytes (B) after incubation with vehicle (white columns) or increasing doses of metformin (black columns) for 24 hours (n = 4 per concentration). Comparisons were performed using repeated measures ANOVA with post hoc Fisher's LSD testing. *, *P* < .05 vs vehicle.

[^3^H]_2_-Cortisol was readily detected in all samples after incubation. Metformin did not increase the conversion of [^3^H]_2_-cortisone to [^3^H]_2_-cortisol in either the hepatocytes or the adipocytes (Figure 3). In both the hepatocytes and adipocytes, the highest metformin concentration (10 mm) decreased cortisol generation by 11βHSD1.

## Discussion

Contrary to our hypothesis, metformin increased whole-body cortisol regeneration by 11βHSD1 in obese men with and without T2DM. This substantial increase in Ra D3-cortisol (∼15%) in both groups suggests that the liver is the most likely tissue responsible because the liver accounts for > 90% of extra-adrenal cortisol production (8, 19). Furthermore, metformin tended to increase conversion of orally administered cortisone to cortisol (a measure of hepatic 11βHSD1 activity) in the ODM group; in one individual, there was, surprisingly, no increase in either circulating cortisone or cortisol concentrations after oral cortisone ingestion, and removal of this subject's data led to a strongly significant increase in hepatic cortisol generation on the metformin phase in the remaining seven subjects (*P* < .01). Adipose tissue and skeletal muscle are alternative tissues that could be responsible, but this is unlikely because the increase in Ra D3-cortisol induced by metformin is greater than the contribution of both tissues combined to whole-body cortisol regeneration under normal conditions (20).

In addition, we have determined that whole-body 11βHSD1 activity is increased in ODM men compared to OND men. There have been conflicting results from previous work examining whether hepatic and whole-body 11βHSD1 activity is altered in T2DM (6, 12, 21); however, these results are consistent with the interpretation that hepatic 11βHSD1 is decreased in euglycemic obesity but not in obesity-associated T2DM (4, 22). Although the ODM group was older, which could be a potential confounder, we have not observed any increase in cortisol regeneration by 11βHSD1 with age in previous studies (6, 16).

We hypothesized that insulin could mediate the effect of metformin on 11βHSD1 because insulin decreases hepatic activity (13). Although it is possible that metformin may have reduced insulin levels in the OND group, there was no suggestion of metformin reducing insulin concentrations in the ODM group, so it is unlikely that insulin drives this regulation, whereas if changes in insulin sensitivity were responsible we may have expected to see a greater effect in the ODM group. Similarly, alterations in glucose concentrations are not responsible because levels were similar during the gliclazide phase without altering 11βHSD1 activity. Our in vitro data suggest that this is not a direct effect, although it is possible that longer incubations may have increased cortisol generation by 11βHSD1. Circulating metformin concentrations are typically 10–40 μm in humans (23), whereas hepatic concentrations can reach 100–200 μm in rodents (24), meaning our in vitro metformin concentrations encompassed the physiologically relevant range. It is possible that the reduction in cortisol conversion at the highest concentration was due to cytotoxicity because metformin has been reported as cytotoxic in the millimolar range, although this is supraphysiological (25).

Recent work has shown that metformin decreases ACTH secretion in humans (26) and reduces ACTH-stimulated adrenal secretion (27). This is consistent with our observation of enhanced peripheral regeneration of cortisol and hence increased negative feedback to the HPA axis; conversely, inhibition of 11βHSD1 results in elevated ACTH (14). However, we did not confirm a reduction in the clearance of cortisol or a decrease in total (adrenal plus 11βHSD1) cortisol production with metformin, although these may be more insensitive measurements.

Our initial hypothesis was that suppression of 11βHSD1 activity by metformin could be the reason for the lack of efficacy of selective 11βHSD1 inhibitors in improving HbA1c (14). However, metformin increased 11βHSD1 activity, an effect that could offset the other metabolic benefits of metformin and potentially enhance any benefit of 11βHSD1 inhibitors. Therefore, this does not appear to be a reason for the lack of efficacy of these drugs.

In conclusion, metformin increases whole-body and likely hepatic regeneration of cortisol by 11βHSD1 in obese men with and without T2DM, so that co-prescription of metformin with selective 11βHSD1 inhibitors may maximize the metabolic benefits of these agents.
